# Functional imaging of immune cell subpopulations in the tumor microenvironment: clinical implications

**DOI:** 10.1172/JCI162962

**Published:** 2022-08-15

**Authors:** Amy B. Heimberger

In cancer, peripheral blood immune monitoring remains an ineffective strategy for assessing immunotherapeutic response, since it does not interrogate immune cell function within the immunosuppressive tumor microenvironment (TME). Tumor immune cells can infiltrate at a high frequency without imposing antitumor activity. Thus, window-of-opportunity clinical trials are an ongoing strategy to characterize the immune cells in the TME. Longitudinal assessments based on the analysis of multiple patient tumor samples collected at several time points would help precisely define a patient’s response to immunotherapy, but in many instances these are unavailable. Furthermore, immune reactivity in the TME can be markedly heterogenous, and limited sampling of the TME can yield misleading results. Therefore, there is a clear clinical need for a noninvasive approach that longitudinally interrogates immune effector responses in the TME. The PET imaging described by Zhou et al. offers such an approach (1).

Prior attempts at imaging TME inflammatory responses with PET have relied on proliferative indicators as a surrogate for activation. For example, [^18^F]-labeled 3′-fluoro-3′-deoxy-thymidine was administered to patients with metastatic melanoma undergoing dendritic cell therapy. Immune responses were visualized in treated lymph nodes soon after therapy and persisted for several weeks (2). Additional PET approaches for tracing activated T cells and quantifying the number of T cells have used [^18^F]F-AraG (3) and ^89^Zr-Df-IAB22M2C (4), respectively. Attempts to develop PET imaging of granzyme B have been ongoing (5, 6), but the study by Zhou et al. is the first to demonstrate its clinical utility (1).

Granzyme B PET could be used to assess various intrinsically induced antitumor immune responses as well as extrinsically administered immune products. For the latter, clinicians could ascertain the status of cytotoxic function and the distribution of adoptively transferred NK cells, T cells, and chimeric antigen receptor T cells within the TME. Granzyme B PET could also be used to determine the response kinetics of immuno-oncology agents such as bispecific T cell engagers, immunomodulatory aptamers, and immune checkpoint inhibitors, and thus help define the timing for combination therapy regimens. PET imaging with improved spatial resolution could also identify tumor regions with a high degree of immune infiltration for follow-up profiling analysis.

With the evolution of any strategy, there will be challenges. For example, NK cells use granzyme B–mediated cytotoxicity early during activation but later use death receptor–mediated cell killing. Furthermore, granzyme B is expressed in immune cell populations not typically associated with proinflammatory responses, such as regulatory B cells, and in Tregs. In cancers for which B cells and Tregs are rare tumor-infiltrating populations, their presence may not necessarily confound PET scan interpretation. Still, with these limitations considered, the current study provides a clear clinical path for interrogating immunological reactivity in the TME using granzyme B PET imaging. The refinement of this imaging approach should improve our understanding of responses to a variety of immunotherapies in patients with cancer.

## Version 1 08/15/2022

Electronic publication
